# Trafficking protein particle complex 6A delta (TRAPPC6AΔ) is an extracellular plaque-forming protein in the brain

**DOI:** 10.18632/oncotarget.2876

**Published:** 2015-02-19

**Authors:** Jean-Yun Chang, Ming-Hui Lee, Sing-Ru Lin, Li-Yi Yang, H. Sunny Sun, Chun-I Sze, Qunying Hong, Yee-Shin Lin, Ying-Tsen Chou, Li-Jin Hsu, Ming-Shiou Jan, Cheng-Xin Gong, Nan-Shan Chang

**Affiliations:** ^1^ Institute of Molecular Medicine, National Cheng Kung University Medical College, Tainan, Taiwan, ROC; ^2^ Departments of Anatomy and Cell Biology, National Cheng Kung University Medical College, Tainan, Taiwan, ROC; ^3^ Department of Pulmonary Medicine, Zhongshan Hospital, Fudan University, Shanghai, PRC; ^4^ Department of Microbiology and Immunology, National Cheng Kung University Medical College, Tainan, Taiwan, ROC; ^5^ Center for Infectious Disease and Signaling Research, National Cheng Kung University Medical College, Tainan, Taiwan, ROC; ^6^ Institute of Basic Medical Sciences, National Cheng Kung University Medical College, Tainan, Taiwan, ROC; ^7^ Department of Medical Laboratory Science and Biotechnology, National Cheng Kung University Medical College, Tainan, Taiwan, ROC; ^8^ Advanced Optoelectronic Technology Center, National Cheng Kung University, Tainan, Taiwan, ROC; ^9^ Institute of Microbiology and Immunology, Chung Shan Medical University, Taichung, Taiwan, ROC; ^10^ Department of Neurochemistry, New York State Institute for Basic Research in Developmental Disabilities, Staten Island, NY, USA

**Keywords:** Alzheimer's disease, protein aggregation, hippocampus, amyloid beta, TIAF1, TRAPPC6A, WWOX, WOX1

## Abstract

Tumor suppressor WWOX is involved in the progression of cancer and neurodegeneration. Here, we examined whether protein aggregation occurs in the brain of nondemented, middle-aged humans and whether this is associated with WWOX downregulation. We isolated an *N*-terminal internal deletion isoform, TPC6AΔ, derived from alternative splicing of the *TRAPPC6A* (*TPC6A*) gene transcript. TPC6AΔ proteins are present as aggregates or plaques in the extracellular matrix of the brain such as in the cortex. Filter retardation assays revealed that aggregate formation of TPC6AΔ occurs preceding Aβ generation in the hippocampi of middle-aged postmortem normal humans. In a *Wwox* gene knockout mouse model, we showed the plaques of pT181-Tau and TPC6AΔ in the cortex and hippocampus in 3-week-old mice, suggesting a role of WWOX in limiting TPC6AΔ aggregation. To support this hypothesis, *in vitro* analysis revealed that TGF-β1 induces dissociation of the ectopic complex of TPC6AΔ and WWOX in cells, and then TPC6AΔ undergoes Ser35 phosphorylation-dependent polymerization and induces caspase 3 activation and Aβ production. Similarly, knockdown of WWOX by siRNA resulted in dramatic aggregation of TPC6AΔ. Together, when WWOX is downregulated, TPC6AΔ is phosphorylated at Ser35 and becomes aggregated for causing caspase activation that leads to Tau aggregation and Aβ formation.

## INTRODUCTION

Accumulated matrix fibrillar β-amyloid (Aβ) plaques and intracellular neurofibrillary tangles (NFT) in the hippocampus has been considered as the hallmark of Alzheimer's disease (AD) [1–3]. These protein aggregates invoke neuronal death and block neurogenesis and learning and memory capabilities in AD patients. Transforming growth factor beta (TGF-β) has been implicated in the AD pathogenesis [4–6]. TGF-β1 causes self-aggregation of TIAF1 (12-kDa TGF-β-induced antiapoptotic factor) *in vitro*, which leads to degradation of membrane amyloid precursor protein (APP) and generation of Aβ and amyloid fibrils [6]. This *in vitro* finding positively correlates with the occurrence of *in vivo* aggregation of TIAF1 in the hippocampi of nondemented, middle-aged humans (age 40–75), and formation of amyloid β, fibrils and plaques in older AD patients (age 70–95) [6]. TIAF1 aggregates are found in degenerative neurons along the interface between metastatic cancer cells and the brain tissue and in the fibrous tissues of lung cancer [7–9].

Here, we report the isolation of an isoform of TRAPPC6A (TPC6A), known as Trafficking Protein Particle Complex 6A. This isoform TPC6AΔ possesses an internal deletion of 14 amino acids at the *N*-terminus. Wild type TPC6A is one of the components in the transport protein particle (TRAPP) complex in yeast [10–14]. In mouse, deletion of *Trappc6a* gene induces a phenotype with mosaic loss of coat pigment [13]. Subunits of TRAPP may exhibit independent functions in specific biological processes in mammals [14]. Human *TRAPPC6A* gene is involved in nonverbal reasoning in 2 Scottish cohorts, and is suggested for a role in AD [15].

We determined whether TPC6AΔ aggregation occurs in the hippocampi of postmortem middle-aged normal humans and AD patients. We showed that TPC6A physically interacted with tumor suppressor WW domain-containing oxidoreductase, designated as human WWOX or FOR, and mouse WOX1 [16–19; Reviews]. WWOX possesses two *N*-terminal WW domains, a *C*-terminal short chain alcohol dehydrogenase/reductase domain (SDR), and a nuclear localization signal in between the WW domains [16–19]. How WWOX blocked TPC6AΔ from self-aggregation, caspase activation and Aβ generation was examined. WWOX participates in embryonic neural development [20], and neuronal injury and damages [21–23]. WWOX inhibits GSK-3β-mediated Tau hyperphosphorylation, and thus prevents AD pathogenesis [24]. Importantly, this event is needed for neuronal differentiation [24]. WWOX and isoform WOX2 are significantly downregulated in the AD hippocampus, and the downregulation leads to increased activities of enzymes in hyper-phosphorylating Tau [24–27].

## RESULTS

### Isolation of an *N*-terminal frame deletion isoform TPC6AΔ in mammalian cells

**Figure 1:**
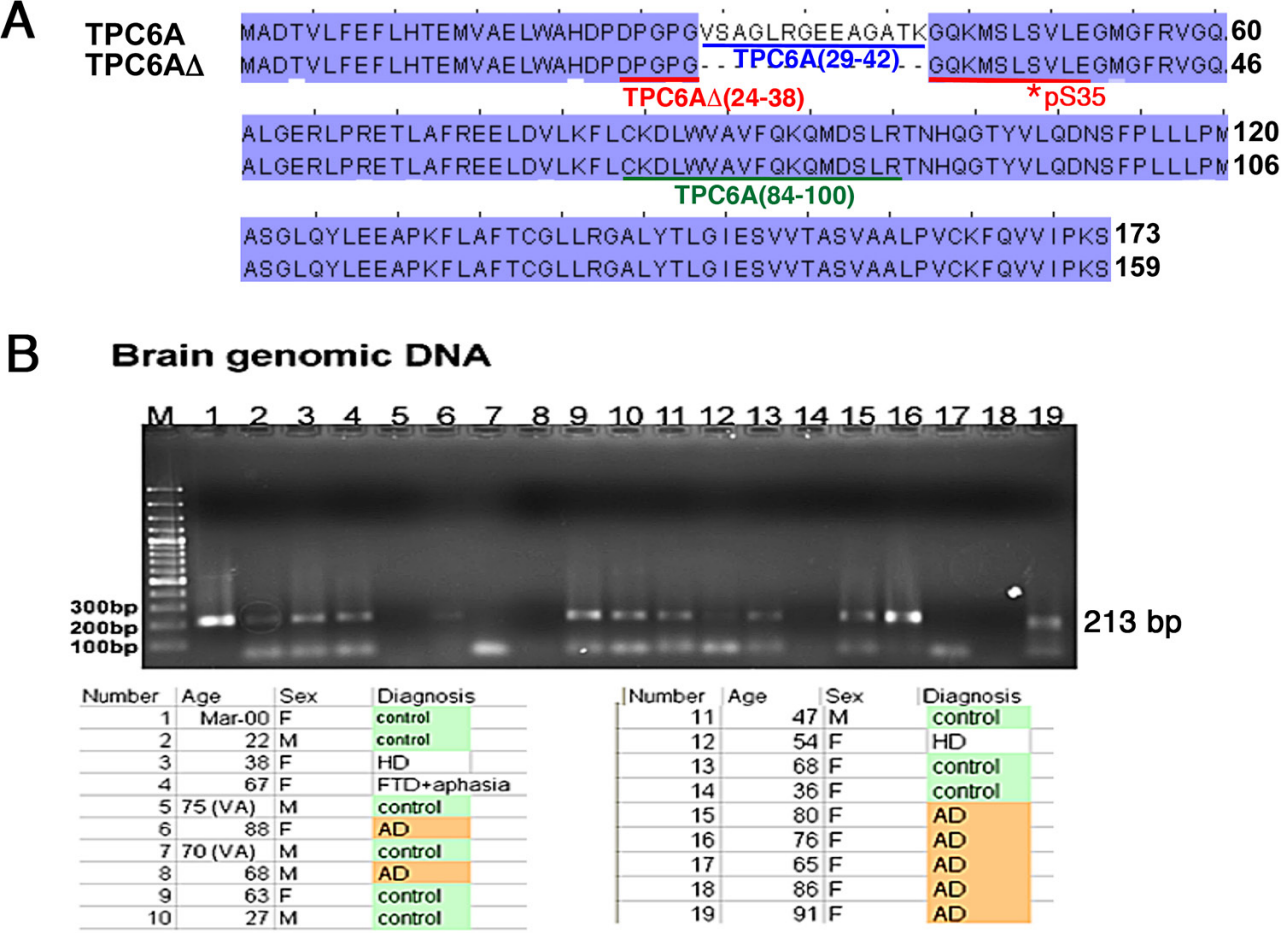
TRAPPC6A isoforms and gene **(A)** Protein sequence alignment: 1) human wild type TRAPPC6A (TPC6A) (GenBank accession NP_077013), and 2) TPC6AΔ with a deletion of 14 amino acids at the *N*-terminus (GenBank accession FJ418644). Two potential phosphorylation sites are Ser49 and Tyr126 for TPC6A, or Ser35 and Tyr112 for TPC6AΔ. Specific polyclonal antibodies were generated in rabbits against the underlined amino acid sequences: TPC6A (29–42) peptide (blue) antibody for wild type protein; TPC6AΔ (24–38) peptide (red) antibody for 17-kDa TPC6AΔ protein; pS35-TPC6AΔ (24–38) (red) peptide antibody for Ser35 phosphorylation of TPC6AΔ; TPC6A (84–100) (green) peptide antibody for wild type and TPC6AΔ. **(B)** Genomic DNA was isolated from indicated postmortem human hippocampal samples. A primer set was designed to amplify the possible deletion in the exon 1 and the flanking areas (total 213 bp, missing region 42 bp, #17949610 to 17949569, Homo sapiens chromosome 19 genomic contig, GRCh37.p5 Primary Assembly), as shown in the mRNA encoding TPC6AΔ. The amplified DNA samples were sequenced and found no deletions. A 90-bp DNA band at the bottom of the gel is a non-specific DNA fragment. “No signal” was due to failure of polymerase chain reaction (PCR).

TPC6AΔ was originally isolated from TGF-β1-treated monocytic U937 cells in a subtraction cDNA library screen. This gene is expressed ubiquitously in many organs and tissues, according to the Genbank database. The deduced protein possesses an internal frame deletion of amino acids #29–42 at the *N*-terminus (Genbank accession FJ418644). The full-length or wild type TPC6A was constructed by inserting a missing DNA frame (42 bases) to the TPC6AΔ cDNA (Figure 1A). Selected amino acid sequence segments for generating specific polyclonal antibodies in rabbits against the full-length TPC6A, TPC6AΔ, and Ser35 phosphorylated TPC6AΔ, respectively, are shown (Figure 1A).

To determine whether the generation of TPC6AΔ mRNA is caused by deletion of exon 1 of human *TRAPPC6A* gene, a primer set was designed to amplify a 213-base region comprising the target DNA segment (42 bp) and the flanking areas at both 5′ and 3′ ends (171 bp). The amplified DNA samples were subjected to sequence determination and shown to be identical to the sequence in the human *TRAPPC6A* gene. Thirty hippocampal samples, including 12 controls and 18 AD patients from postmortem Caucasians, were examined. None of the genomic DNA samples were deleted in the exon 1 of human *TRAPPC6A* gene (Figure 1B). Similar results were observed by examining 50 genomic DNA samples in a random Asian population in Taiwan (data not shown).

**Figure 2:**
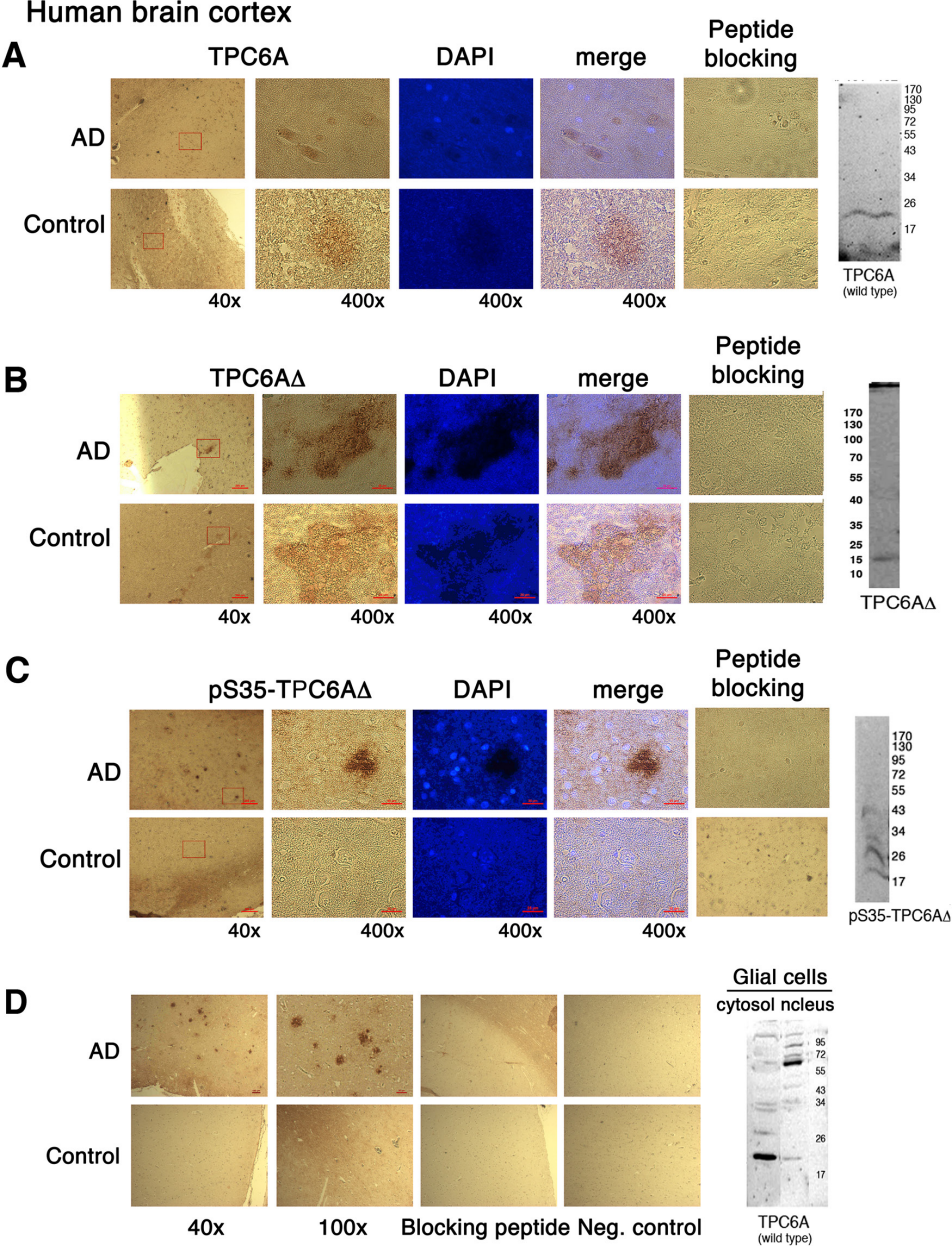
TPC6AΔ aggregates in the human brain cortex Human brain cortical tissue sections from AD patients and age-matched controls, along with lysates from cell lines, were used for IHC and Western blotting, respectively: **(A)** wild type 20-kDa TPC6A in human brain sections and wild type Wwox MEF cells stained with the wild type specific TPC6A (29–42) peptide antibody; **(B)** 17-kDa TPC6AΔ in human brain sections and SK-N-SH cells stained with the TPC6AΔ (24–38) peptide antibody; **(C)** pS35-TPC6AΔ in human brain sections and SK-N-SH cells stained with the pS35-TPC6AΔ (24–38) peptide antibody; **(D)** wild type and TPC6AΔ in human brain sections and mouse glial cells stained with the pan-specific TPC6A (84–100) peptide antibody. Nuclei were stained with DAPI. In controls, immunizing peptides were used to block the immunoreactivity. Also, in negative controls (see D), no primary antibodies were used in the IHC. Enlargements were made from boxed areas at 40x magnification to 400x. Scale bars for 40x, 100x, and 400x are 200, 100, and 25 μm, respectively.

Based upon the aforementioned observations, we determined whether *TRAPPC6A* mRNA undergoes alternative splicing. Computational analyses using 1,400 genomic sequences starting from the CDS sequence on *TRAPPC6A* exon 1 were performed [28, 29]. Results from 2 different web-based tools (NNSplice and NetGene) all predicted that the nt position 85 can be used as an alternative 5′ donor site to initiate splicing and leads to a 42-bp deletion on *TRAPPC6A* exon 1 sequence. Additional evidence from EST database searching showed that 13 TPC6A cDNAs out of 55 in total in humans are with the 42-bp deletion. Proteins corresponding to wild type TPC6A and TPC6AΔ are shown in cells (Figure 2), which supports the occurrence of alternative splicing of *TRAPPC6A* mRNA. Data are provided to show the production of our homemade antibodies (Supplementary Figure 1). Also, the validity and protein aggregation are shown under various experimental conditions (Supplementary Figure 2 and 3).

### TPC6AΔ aggregates in human AD hippocampal and cortical tissues

Unlike in the yeast [10–12], the functional properties of mammalian TPC6A are largely unknown. By immunohistochemistry (IHC), we showed the presence of extracellular TPC6AΔ aggregates or plaques with phosphorylation at Ser35 in the human AD cortex (Figure 2B–2D). In comparison, much less aggregation was shown for the wild type TPC6A in the brain cortex in age-matched control samples (Figure 2A). Specific antibody for the wild type TPC6A was used (Figure 2A). Pan specific antibody against both wild type and TPC6AΔ was also used to demonstrate aggregates in the cortex of AD patients (Figure 2D). Our antibody is specific, as each immunizing peptide blocked the corresponding immunoreactivity (Figure 2A–2D). By Western blotting, the 20-kDa wild type protein was identified by the specific antibody (duplicate loading; Figure 2A). Antibodies against TPC6AΔ and its phosphorylated form probed the 17-kDa TPC6AΔ and polymerized pS35-TPC6AΔ, respectively, in the neuroblastoma SK-N-SH cells (Figure 2B–2C). As determined using the pan-specific antibody, cytosolic wild type TPC6A is shown in the glial cells (Figure 2D). However, TPC6A became polymerized in the nucleus (Figure 2D).

### Aggregation of TPC6A and TIAF1 in nondemented human hippocampi

**Figure 3:**
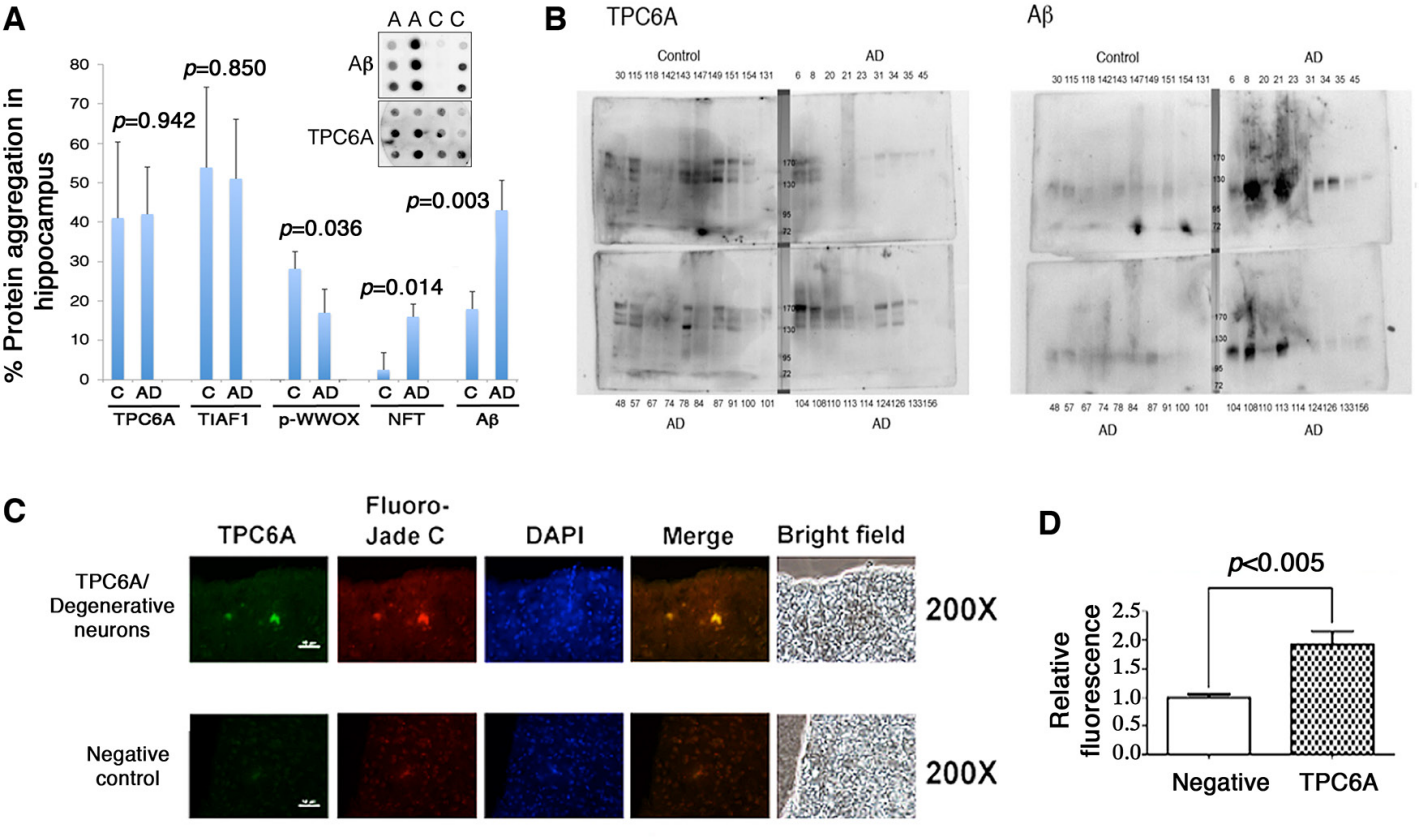
Soluble and insoluble TPC6A aggregates are present in the hippocampi of nondemented human controls and AD samples **(A)** Frozen postmortem AD and control human hippocampal tissues were homogenized by sonication. After centrifugation, the insoluble pellets were further extracted and analyzed by filter retardation assay. Western blotting was then carried out using specific antibodies for TPC6A (pan-specific), TIAF1, Tyr33-phosphorylated WWOX (p-WWOX), NFT and Aβ. No significant difference was shown in TPC6A or TIAF1 aggregation for samples between younger nondemented controls (14 samples per group; total 3 groups; 42 to 76 years old) and older AD patients (24 samples per group; total 4 groups; 71 to 90 years old). C = control; A or AD = Alzheimer's disease. **(B)** The supernatants of the aforementioned samples were analyzed by 7% non-reducing SDS-PAGE. Western blotting analyses show the presence of soluble aggregates of TPC6A and Aβ in the representative protein preparations. Sample numbers are shown in each panel. Molecular weight markers: 170, 130, 95, 72 kDa. **(C)** Hippocampal tissue sections of APP/PS1-transgenic mice were stained with aliquots of TPC6A pan-specific antiserum and then with an Alexa Fluor 488-conjugated secondary antibody. Fluoro-Jade C was used for staining degenerative neurons, and nuclei stained with DAPI. TPC6A aggregates are localized in the degenerative neurons. A representative tissue section is shown. Scale bar: 50 μm. In negative controls, the sections were stained with the secondary antibody only, followed by staining with Fluoro-Jade C and DAPI. **(D)** Immuno-intensity was measured and normalized to negative controls (mean ± standard deviation, *n* = 5; **p < 0.05, student's *t* test).

By filter retardation assay, we have recently demonstrated the presence of water-insoluble TIAF1 aggregates in the hippocampi of nondemented humans at 40–75 years old, and the aggregates possess increasing amounts of Aβ in the older AD samples (70–95 years old) [6]. Also, TIAF1 aggregation leads to the formation of Aβ *in vitro* [6]. Here, water-insoluble TPC6A aggregates were found in the hippocampi of postmortem humans, as determined by filter retardation assay (Figure 3A). Total samples from nondemented, younger controls (59±17 years old; *n* = 42) and older AD patients (80±8.8 years old; *n* = 96) were randomly divided into 3 and 4 groups, respectively. TPC6A aggregates were found in both control and AD groups to a similar extent (~40% positive) (Figure 3A), suggesting that TPC6A aggregates are relatively stable in the brain. Similar results were observed with TIAF1 aggregates [6]. Both wild type TPC6A and TPC6AΔ are present in the aggregates, as determined using the TPC6A (84–100) peptide antibody. In agreement with our previous reports [6, 25], the levels of protein aggregates for Tyr33-phosphorylated WWOX (p-WWOX) were significantly reduced by ~40% in the AD samples, compared to nondemented controls (Figure 3A). The extent of protein aggregation for NFT and Aβ was significantly increased in the AD samples, compared with the nondemented controls (Figure 3A).

Next, we determined the presence of water-soluble aggregates using the aforementioned control and AD hippocampal samples by non-reducing SDS-PAGE and Western blotting. Large-sized TPC6A aggregates are shown in both nondemented and AD hippocampi (Figure 3B). Aβ aggregates are more abundant in the AD hippocampal samples than in the controls (Figure 3B). We also showed the significantly increased levels of TPC6A aggregates in the degenerative neurons of hippocampi of APP/PS1-transgenic mice, as determined by immunofluorescence microscopy (Figure 3C–3D).

### TGF-β1-induced TPC6AΔ aggregation for leading to caspase 3 activation and Aβ production

**Figure 4:**
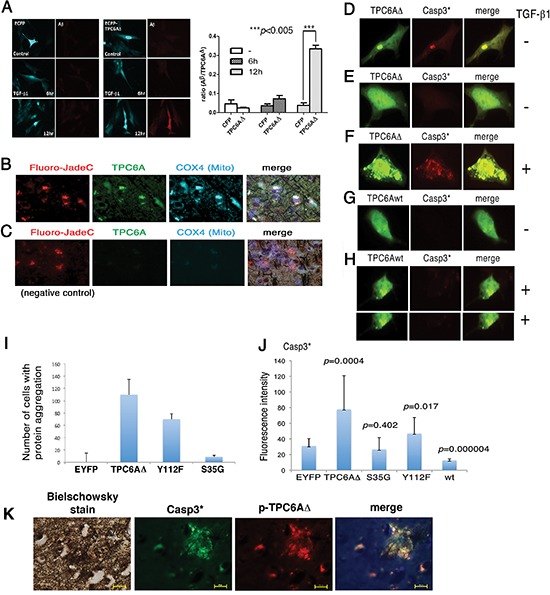
TGF-β1 induces TPC6AΔ aggregation for leading to caspase activation **(A)** Neuroblastoma SK-N-SH cells were transiently expressed with ECFP or ECFP-TPC6AΔ and treated with TGF-β1 (5 ng/ml) for indicated times, followed by fixation, permeabilization and staining with Aβ antibody. The ratios of the expression of Aβ to TPC6AΔ are shown (*n* = 5). **(B)** TPC6A aggregates (green) are present in the mitochondria of degenerative neurons in the AD hippocampus. Tissue sections were pre-stained with the Bielschowsky stain (brown to dark), and then stained with Fluoro-Jade C Red for degenerative neurons (red), specific COX4 antibody for mitochondria (cyan), DAPI for nuclei (blue) and pan-specific antibody for TPC6A (green). 600x magnification; Scale bar: 10 μm. **(C)** In negative controls, no primary antibody was used in the immunostaining. **(D)** Transiently overexpressed EYFP-TPC6AΔ was aggregated in SK-N-SH cells, and caspase 3 became activated (Casp3*). **(E)** No caspase 3 activation was observed, if ectopic TPC6AΔ was not aggregated. **(F)** TGF-β1 increased TPC6AΔ aggregation and caspase 3 activation. **(G–H)** Wild type TPC6A (TPC6Awt), either in a native or an aggregated state, did not induce caspase 3 activation. **(I–J)** SK-N-SH cells were transiently overexpressed with the indicated expression constructs. The number of cells harboring protein aggregation was counted, and the extent of caspase 3 activation was measured (*n* = 3; Student's *t* tests; ~50 cells counted per experiment). **(K)** Colocalization of S35-phosphorylated TPC6AΔ with activated caspase 3 in the AD hippocampal tissue sections. A representative data is shown.

We determined whether aggregated TPC6AΔ causes caspase activation. Neuroblastoma SK-N-SH cells were transiently overexpressed with ECFP or ECFP-TPC6AΔ. After culturing for 24 hr, the cells were treated with TGF-β1 (5 ng/ml) for 6–12 hr. TGF-β1 significantly increased the production of Aβ in the ECFP-TPC6AΔ-expressing cells in 12 hr (Figure 4A). We investigated the possible presence of aggregated TPC6A in the degenerative neurons. By staining AD hippocampal tissue sections, we showed the presence of TPC6A in the mitochondria of degenerated neurons (Figure 4A). Degenerative neurons were stained with Fluoro-Jade C [6], mitochondria with COX4 antibody, and TPC6A with the pan specific antibody. In the negative controls, no primary antibodies were used (Figure 4C). Presence of TPC6A in the mitochondria suggests that TPC6A may induce caspase activation during neuronal death.

Transiently overexpressed wild type TPC6A or TPC6AΔ was equally potent in causing cell death (~50–75% apoptosis of SK-N-SH and other cell lines using 10 μg expression constructs). When ectopic TPC6AΔ became aggregated in SK-N-SH cells, TPC6AΔ induced caspase 3 activation (Figure 4D). Without aggregation, TPC6AΔ did not induce activation of caspase 3 (Figure 4E). TGF-β1 increased TPC6AΔ aggregation and subsequent caspase 3 activation (Figure 4F). In contrast, overexpressed wild type TPC6A, with or without aggregation, did not induce caspase 3 activation (Figure 4G–4H).

By site-directed mutagenesis, the S35G mutant of TPC6AΔ significantly lost its capability in aggregation and did not activate caspase 3 (Figure 4I–4J). The Y112F mutant had a reduced effect in aggregation and causing caspase 3 activation (Figure 4I–4J). Overexpressed wild type TPC6A underwent aggregation and caused apoptosis, but did not induce caspase 3 activation (Figure 4G–4J). In negative controls, EYFP did not undergo aggregation when overexpressed (Figure 4I–4J). S35-phosphorylated TPC6AΔ was shown to colocalize with activated caspase 3 in the human AD hippocampal tissue sections (Figure 4K).

### *Wwox* gene ablation induces TPC6AΔ and tau aggregation in the brain of 3-week-old knockout mice

**Figure 5:**
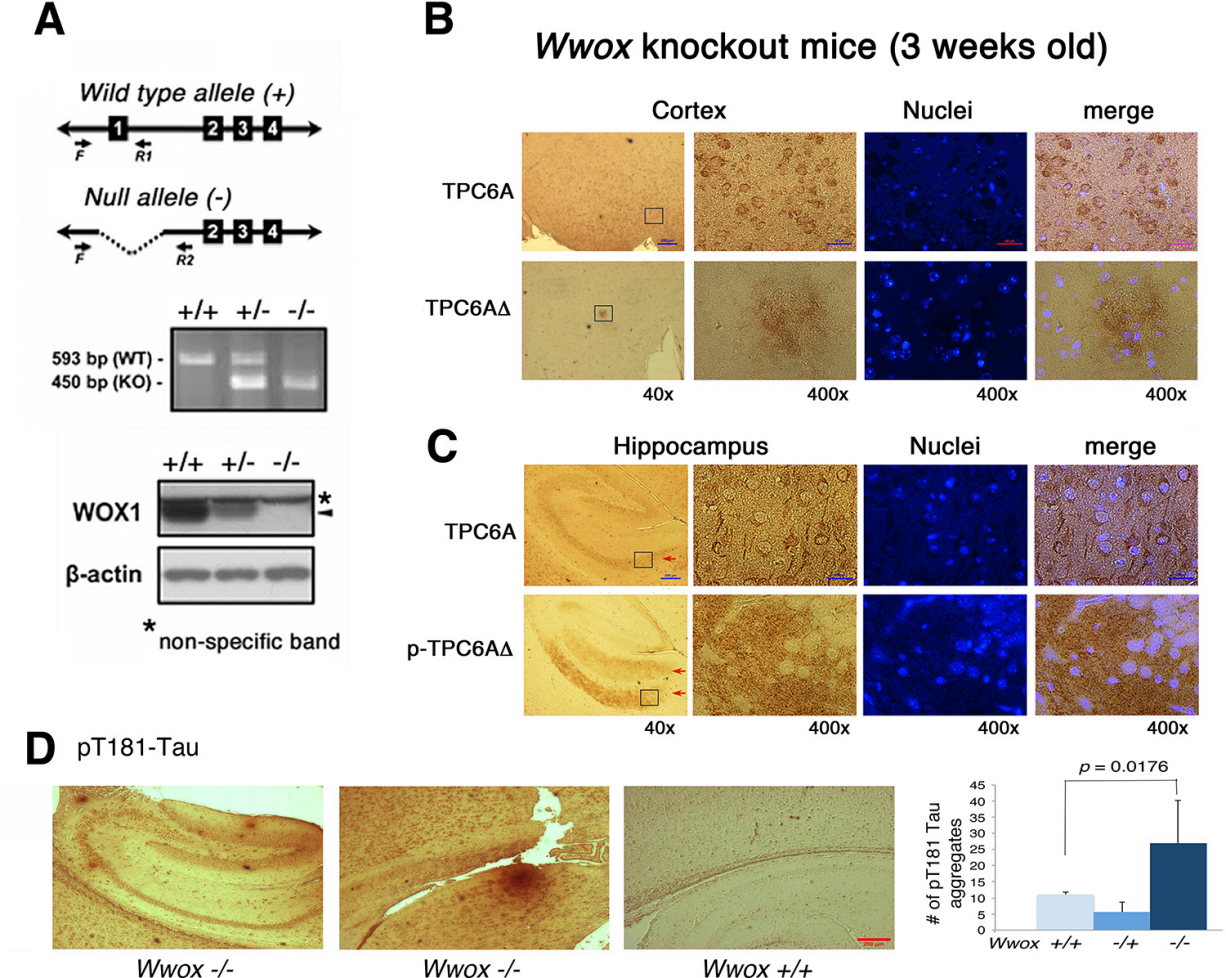
TPC6AΔ is an extracellular protein aggregate **(A)** Ablation of *Wwox* gene at the exon 1 was carried out in mice. Genotyping of the generated animals and the expression of mouse WWOX/WOX1 protein (see arrow head) in MEF cells are shown. **(B)** The wild type TPC6A is mainly expressed in the perinuclear areas of neurons in the cortex of 3-week-old knockout mice. However, TPC6AΔ exhibits as extracellular aggregates in the brain cortex. **(C)** TPC6A is localized in the cytoplasm of neurons in the pyramidal layer of the hippocampus (see arrow). pS35-TPC6AΔ and TPC6AΔ are present in the extracellular matrix of adjacent stratum oriens and stratum radiatum as aggregates (see red arrows; data not shown for TPC6AΔ). Enlargements were made from boxed areas at 40x magnifications to 400x. Scale bars for 40x and 400x are 200 and 25 μm, respectively. **(D)** The numbers of pT181-Tau aggregates were counted in the brain hemisphere sections of *Wwox* +/+. −/+, and −/− mice (*n* = 5). Two representative pictures are shown for the brain tissue sections of Wwox −/− mice, and one for Wwox +/+ mice.

We have demonstrated that WWOX is significantly downregulated in the hippocampi of AD patients [24–26]. To evaluate the physiological significance, we generated *Wwox* gene knockout mice. Genotyping of the generated animals is shown (Figure 5A). The mice can only survive for about a month, which is in agreement with a previous report [30]. The wild type TPC6A is mainly expressed in the perinuclear areas of neurons in the brain cortex of 3-week-old knockout mice (Figure 5B). In contrast, TPC6AΔ aggregates are expressed in the extracellular matrix of the cortex (Figure 5B). Wild type TPC6A is expressed in the cytoplasm of neurons of the pyramidal layer of the hippocampus (see arrow; Figure 5C). However, pS35-TPC6AΔ and TPC6AΔ are located in the adjacent stratum oriens and stratum radiatum, and the proteins appear as extracellular aggregates (see arrows, Figure 5C; data not shown for TPC6AΔ). Similarly, in cerebellum, wild type TPC6A is localized in the cytoplasm of Purkinje cells, whereas pS35-TPC6AΔ and TPC6AΔ are expressed in the matrix of white matter (data not shown). In parallel, pT181-Tau aggregates were significantly increased in the brain hemisphere sections of the 3-week-old *Wwox* knockout mice, as compared to wild type and heterozygous mice (Figure 5D).

### Knockdown of WWOX by siRNA induces aggregation of TPC6AΔ and TIAF1

**Figure 6:**
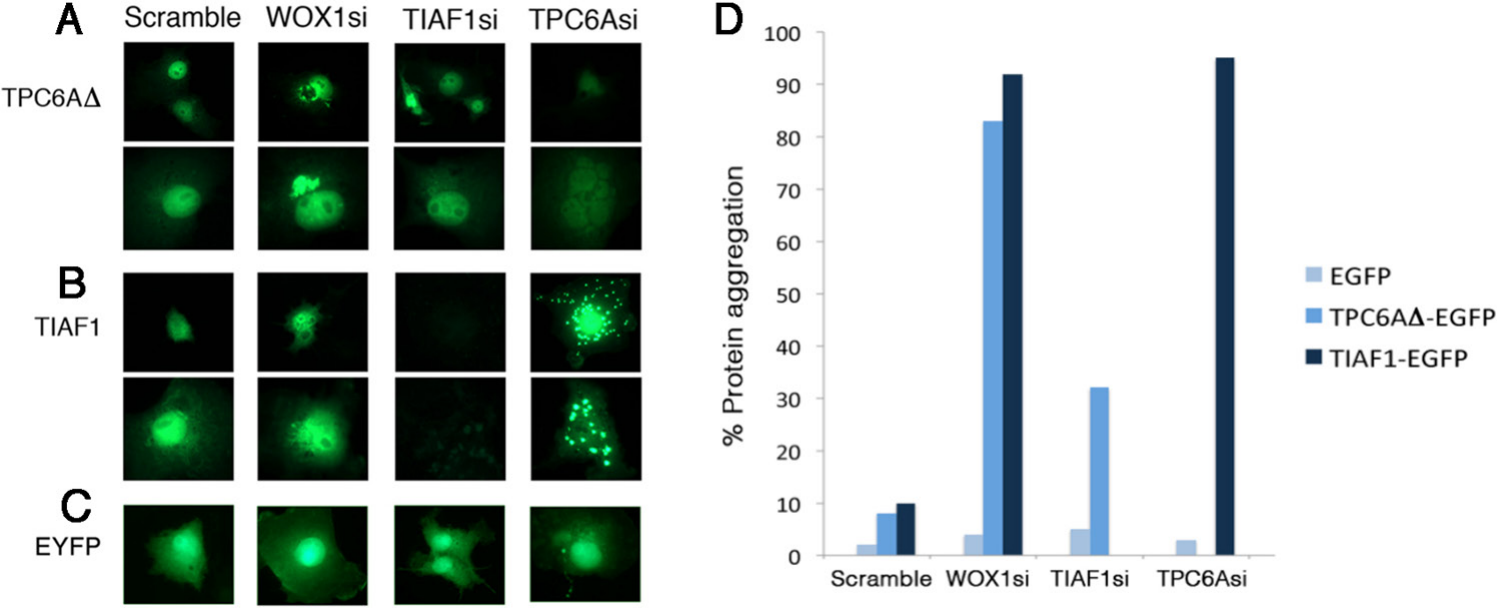
Induction of TPC6A and TIAF1 aggregation upon knockdown of endogenous WOX1 by siRNA **(A–C)** COS7 cells were transfected with expression plasmid constructs for EYFP-TPC6AΔ, EYFP-TIAF1, or EYFP, in the presence of one of the siRNA-expressing constructs, including scramble, WOX1si, TIAF1si and TPC6Asi. 24 hours later, protein aggregation was examined by fluorescent microscopy (~150 cells counted). Data are shown in duplicates for (A) and (B). **(D)** Tabulated data is shown.

In parallel experiments, we examined the effect under WWOX knockdown. COS7 cells were co-transfected with small interfering RNA (siRNA)-targeting WWOX and EYFP-TPC6AΔ, EYFP-TPC6A, or EYFP. The cells were then cultured for 24 hr. When WWOX was knocked down by siRNA (WOX1si), ectopic TPC6AΔ and TIAF1 became aggregated by greater than 80% of cells (~100 cells counted; Figure 6A–6D). In appropriate controls, no aggregation (0%) was observed with EYFP alone in the presence of WOX1si or WWOXsi or scramble. Also, when cells were transfected with a “scramble DNA” construct, less than 10% protein aggregation was shown for TIAF1 and TPC6A (Figure 6A–6D). By time-lapse FRET microscopy, generation of cytosolic TPC6AΔ aggregates (see puncta) occurred when COS7 cells were transfected with WWOXsi (Video 1). However, no protein aggregation occurred when cells were transfected with a scrambled construct (Video 2). Together, the aforementioned observations are in parallel with the results from the mouse *Wwox* knockout model, suggesting that without WWOX *in vitro* and *in vivo*, TPC6A and TIAF1 start to polymerize or aggregate.

## DISCUSSION

In summary, by utilizing specific antibodies, we have demonstrated for the first time that wild type TPC6A and isoform TPC6AΔ are expressed in distinct brain areas. TPC6AΔ is a plaque-forming protein in the brain extracellular matrix, whereas wild type TPC6A is a cytosolic protein. TPC6A, for instance, is expressed in the pyramidal layer and TPC6AΔ in the adjacent molecular layer of the hippocampus. TPC6AΔ, but not the wild type, forms cortical plaques. TPC6A is abundant in the Purkinje cells of cerebellum, but TPC6AΔ is polymerized in the white matter. Most strikingly, TPC6AΔ and Tau plaques can be found in the brain cortex of *Wwox* knockout mice of less than 3 weeks old. WWOX is frequently downregulated in the hippocampi of AD patients [25, 26], suggesting that WWOX is crucial in preventing the aggregation of TPC6AΔ and Tau. We determined that TGF-β1 causes dissociation of WWOX from TPC6AΔ, thus leading to the aggregation of TPC6AΔ and TIAF1 and subsequent events including activation of caspases, and Aβ production. In parallel, knockdown of WWOX causes aggregation of TIAF1 and TPC6AΔ. Apparently, TPC6AΔ contributes a critical role in the aggregation of neuronal proteins and neurodegeneration.

In agreement with our previous observations [6], we determined that TPC6AΔ/TIAF1 aggregates or plaques are present in the hippocampi of normal individuals at mid-ages, and the complexes possess increasing amounts of Aβ in the hippocampi of older AD patients. A critical finding from our study is that aggregating TPC6AΔ activates caspases and contributes, in part, to Aβ generation. Thus, the ratio of wild type TPC6A versus TPC6AΔ isoform is likely to determine the tendency of AD pathogenesis in normal individuals. Caspase activation is known to contribute, in part, to the breakdown of APP and formation of Aβ [31].

Whether TGF-β1 regulates the binding of WWOX with TPC6AΔ is unknown and is being determined in this laboratory. Previously, we determined that TGF-β1 induces the relocation of WWOX from the cytoplasm to the nucleus [32] and causes TIAF1 self-aggregation, Aβ generation and apoptosis in certain cells [6]. Conceivably, WWOX is likely to complex with TPC6AΔ, and TGF-β1 would effectively increase their accumulation in the nucleus.

It is reasonable to assume that TPC6A aggregates serve as the nucleation sites for TIAF1 binding, caspase activation, and deposition of Aβ. Alteration of Ser35 to Gly35 in TPC6AΔ significantly abolishes the capability of TPC6AΔ in activating caspase 3. TPC6AΔ plaques are mainly phosphorylated at Ser35 in the brain. These findings indicate that phosphorylation of TPC6AΔ is essential for causing neurodegeneration. In addition, TPC6AΔ/TIAF1 aggregation is shown in hippocampi of both postmortem AD patients and non-demented controls. The complexes appear to be stable in the brain, since their presence is found in the hippocampi of mid-aged normal individuals and older aged AD patients. TPC6A/TIAF1 aggregates complex with Aβ deposits in the AD hippocampus, again suggesting that the aggregates are nucleation sites for Aβ.

WWOX is frequently downregulated in the hippocampus of AD patients [25]. We found that knockout *Wwox*−/− MEF cells are prone to possess aggregates of TPC6A, TIAF1, JNK1 and upregulated expression of β-secretase and Tau tangles (data not shown), suggesting a role of WWOX in stabilizing proteins and blocking their aggregation. Importantly, we demonstrated the presence of TPC6A plaques and pT181-Tau aggregates in the cortex of *Wwox*−/− mouse brain. *Wwox*−/− mice can only survive for one month. That is, plaques quickly form in less than a month. Recently, we demonstrated that WWOX physically interacts with GSK-3β, and thereby suppresses Tau hyperphosphorylation [24]. WWOX also binds MEK1 for preventing the activation of ERK, thus reducing ERK-mediated phosphorylation of Tau [33]. Together, from previous and this studies, we have provided strong evidence for a crucial role of WWOX in preventing protein aggregation and neurodegeneration.

TPC6A is a subunit of TRAPP complex in yeast and mammals. In yeast, TPC6A is localized in ER and Golgi. In contrast, TPC6A is mainly localized in nuclei and the perinuclear regions in mammalian cells. The functional role TPC6A in the nuclei is unknown. A leucine zipper motif is predicted near the *C*-terminal of TPC6A, suggesting that TPC6A might be a DNA-binding protein. We predicted two possible phosphorylation sites in TPC6AΔ, Ser35 and Tyr112. Alteration of Ser35 to Gly35 abolishes TGF-β-mediated aggregation of TPC6AΔ. The Ser35 phosphorylation was verified by our produced antibody. Alteration of Tyr112 to Phe112 slightly reduces TPC6AΔ aggregation (<30%) in the presence or absence of TGF-β. Importantly, overexpressed wild type TPC6A may undergo aggregation, but fails to activate caspases. Binding of TPC6AΔ with wild type TPC6A is weak, and may not cause generation of caspase activation (data not shown). However, both proteins become aggregated in the nucleolus upon stimulation with TGF-β1.

WWOX prevents hyperphosphorylation of Tau by inhibiting GSK-3β, ERK and other kinases, thereby preventing the formation of NFTs in neurons [24, 25]. Interestingly, blocking of GSK-3β activity by WWOX enhances neurite outgrowth and neuronal differentiation [24]. WWOX also binds Tau via its *C*-terminal SDR domain, whereas how the binding regulates the hyperphosphorylation of tau is unknown. The likely scenario is that WWOX may act as a chaperone, which stabilizes proteins from misfolding and being degraded by the ubiquitin/proteasome system.

## MATERIALS AND METHODS

### Cell lines, chemicals and human postmortem hippocampal tissues

Cell lines used in these studies were monkey kidney COS7 fibroblasts, human neuroblastoma SK-N-SH cells (American Type Culture Collection), and isolated primary rat glial cells. Human postmortem frozen hippocampal tissues, as well as fixed tissue sections from hippocampi, were obtained from the Department of Pathology, University of Colorado Health Sciences Center (by Dr. CI Sze, before 2005) [6, 25]. IRB approval was waived. Informed consents were obtained from the family members of the deceased patients. Frozen hippocampal sections of APP/PS2 transgenic mice were prepared as described [6]. Fluoro-Jade C Red was from Chemicon/Invitrogen. The full length Zfra peptide was synthesized (GeneMed Synthesis).

### cDNA expression constructs, mutant clones, and transient expression

TPC6AΔ was constructed in pECFP-C1, pEGFP-C1 and pEYFP-C1 (Clontech), respectively. The primers for these vectors were designed using a Mac DNASIS software (Hitachi), as follows: Forward primer, 5′-TCGAATTCTATGGCGGATACTGTGT; Reverse primer, 5′-ACGAATTCGATTAGGATTT CGGAATCAC. Site-directed mutagenesis was also employed to alter conserved phosphorylation sites Tyr112 and Ser35 using the following primer sets: 1) S35G, forward 5′-ATGAG CCTGGGAGTCCTGGA and reverse 5′-AGGTCCTGAGGGTCCGAGTA; 2) Y112F, forward 5′-TGGCCTGCAGTTTCTGGAGG and reverse 5′-ACCGGACGTCAAAGACCTCC. Expression constructs of full-length and truncated WWOX or WOX1 were made [6, 32]. Transient overexpression of the indicated expression constructs in cells was performed by electroporation (2×10^6^ cells; 200V, 50ms; BTX ECM 830 Square Wave Electroporator) or using GeneFector (Venn Nova), as described [6, 34].

### Computational analysis

To predict the possibility of alternative splicing on exon 1 of *TRAPPC6A* gene, online software developed with different algorithms was applied. The tools included Splice Site Prediction by Neural Network (NNSplice; http://www.fruitfly.org/seq_tools/splice.html) [35], and NetGene2 (http://www.cbs.dtu.dk/services/NetGene2/) [36].

### Isolation of TPC6AΔ cDNA and construction of full-length TPC6A

We constructed a cDNA library by treating human monocytic U937 cells with TGF-β1. The TPC6AΔ cDNA was isolated from this library (with deletion at amino acids #29–42; GenBank accession FJ418644). Full-length TPC6A-pEGFPC1 DNA was constructed by inserting synthetic primers into the TPC6AΔ cDNA. Primers, containing the deleted sequence of the 5′-end truncation in TPC6AΔ, were designed: forward, 5′-CCGACCCCGGCCCGGGGG**TGAGCGCCGGGCT CCGTGG GGAGGAAGCGGGGGCCACCAAGG** GACAGAAGATGAGCCTG; reverse, 5′-CAGGC TCATCTTCTGTC**CCTTGGTGGCCCCCGCTTCCTC CCCACGGAGCCCGGCGCTCA**CCCCCGGGCCGG GGTCGG. At the 5′ end, the primer has 18 bases corresponding to the TPC6Awt sequence, followed by the deleted 42 bases (bold) and then 17 bases for the 3′ end. PCR was performed under the following cycling condition: step 1) heating at 94°C for 45 sec, step 2) cycling for 25 times at 94°C for 45 sec, 65°C for 45 sec, and 72°C for 12 min, and 3) final synthesizing at 72°C for 10 min. The PCR product was digested with DpnI (New England BioLabs) at 37°C for 3 hr to remove the original templates, and the amplified cDNA was transformed to *E. coli* Top 10 (Invitrogen). Positive clones were isolated and identified by sequencing analysis.

### Antibodies and antibody production in rabbits

Use of rabbits for antibody production was approved by the Institutional Animal Care and Use Committee of the National Cheng Kung University Medical College. Four TPC6A or TPC6AΔ peptides were synthesized (Figure 1A): 1) **C**KDLWVAVFQKQMDSLR, amino acid #84–100 for pan-specific antibody production; 2) **C**DPGPGGQKMSLSVLE, amino acid #24–38 for antibody against TPC6AΔ; 3) **C**DPGPGGQKMSL**^p^**S****VLE, amino acid #24–38 with phosphorylation at Ser35 for antibody against p-TPC6AΔ; 4) **C**VSAGLRGEEAGATK, amino acid #29–42 for antibody against wild type TPC6A. These peptides were conjugated with keyhole limpet hemocyanin (KLH) for antibody production in rabbits (Antibody Production and Purification kit from Pierce/Invitrogen) [6, 20, 25, 27, 34]. The *N*-terminal cysteine in each peptide sequence was added to covalently conjugate with KLH. Specificity of the antisera was tested using the synthetic peptides to block immunostaining. Commercial antibodies used were against: Aβ (AbD Serotec Cat# MCA2172, RRID:AB_323833), APP (EMD Millipore) [6, 20, 25], NFT (neurofibrillary tangles; Invitrogen) [25], α-tubulin (Sigma-Aldrich), activated caspase 3 (Cell Signaling Technology), and TIAF1 (Abcam) [6, 37]. Antibodies against WWOX and Tyr33-phosphorylation in WWOX were made and purified as described [20, 25, 27, 32]. Adobe Photoshop CS5 software was used to analyze the extent of protein expression from Western blots.

### Immunohistochemistry, immunofluorescence, and time-lapse fluorescence microscopy

De-parafinization, immunohistochemistry, and immunofluorescence staining were performed as described [6, 20–23, 34, 38, 39].

### Filter retardation assay and protein aggregation assay

Filter retardation assay was performed using postmortem human frozen hippocampal extracts [6]. Briefly, samples were homogenized in a protein lysis buffer, and centrifuged in a microfuge (13,200 rpm for 30 min at 4°C). The pellets were harvested and added 100 μl DNase I buffer (20 mM Tris-HCl, pH 8.0, 15 mM MgCl_2_, 1 mg/ml DNase I), and then incubated for 1 hr at 37°C. Protein preparations were quantified (BCA assay kit, Pierce). Each sample containing 10 μg, 30 μg and 60 μg protein, respectively, was diluted in 100 μl sample buffer (1% SDS, 8% β-mercaptoethanol in PBS), boiled for 10 min at 95°C, and then filtered through 0.2 μm cellulose acetate membranes using a dot-blot apparatus (BioRad). Each well was washed by 200 μl 0.1% SDS twice. Then, the membranes were analyzed by standard Western blotting to determine the presence of Aβ, TPC6A and other proteins of interest.

### Development of *Wwox* knockout mice

Generation of gene-targeting constructs and mouse chimera was performed at the Transgenic Mouse Models Core Facility, National Core Facility Program for Biotechnology at the National Taiwan University Medical College, Taipei (http://140.112.133.74/). Briefly, we have designed insertion of LoxP sites to a vector for targeting exon 1 and 2/3/4, respectively, by recombineering technology [40] (Supplementary Figure 4A–4B). Cre-mediated recombination of sequences flanked by LoxP sites was performed in embryonic stem (ES) cells [41]. The targeted ES cell clones were selected and injected into blastocysts to generate chimeric mice. Chimeras were crossed with C57BL/6 mice to obtain germline transmission of the targeted allele. Heterozygous mice were then interbred to obtain wild-type *Wwox*^+/+^, heterozygous *Wwox*^+/−^, and homozygous knockout *Wwox*^−/−^ mouse embryos. Mouse embryonic fibroblasts (MEF) were established from ~E16.5 mouse fetuses. PCR genotyping was performed using primer sequences (5′-tgagcttgggagaagtgggtactttg, 5′-agctctatactatactggctggctgg, and 5′-aggtgttggaga cttctccactgcta for exon 1-deletion; 5′-gctctgtgaga ccatttggacagtgt, 5′-cttgattctgctgcctctgcttccta, and 5′-cgagagaaggaagcctgttatctaga for exon 2/3/4-deletion) specific for the wild type or the targeted allele. All experimental procedures were carried out in accordance with an approved protocol for animal use from the Institutional Animal Care and Use Committee of National Cheng Kung University.

### PCR analysis of partial exon 1 deletion in human TRAPPC6A gene

To determine possible frame deletion in *TRAPPC6A* gene, a primer set was designed to amplify a 213-base region flanking a genomic DNA segment (42 bases lacking in the TPC6AΔ cDNA) and a portion of non-deleted areas at both 5′ and 3′ ends: forward, 5′-GTTTCTTCACACGGAGATGG (in exon 1), and reverse, 5′-CCACTTTCCAAAGGAGGAAG (in intron 1–2). The optimal annealing temperature is 54–55^o^C, as determined by gradient PCR (Mastercycler gradient, Eppendorf). The possibly deleted region in chromosome 19q13.32 is 5′-GTGAGCGCCGGGCTCCGTGGGGA GGAAGCGGGGGCCACCAAG, encoding amino acid #29–42 in the wild type TPC6A. Genomic DNA samples were purified from human normal and AD hippocampal tissues. The amplified DNAs were subjected to sequence determination.

### Data analysis

Where indicated, all experiments were performed 3–5 times. Data were presented as mean ± standard deviation. Student's *t*-tests were carried out for statistical analysis.

## SUPPLEMENTARY FIGURES


