# Supplementary material for: Trafficking protein particle complex 6A delta (TRAPPC6AΔ) is an extracellular plaque-forming protein in the brain
Source: Oncotarget. 2015 Feb 19;6(6):3578–89. doi: 10.18632/oncotarget.2876 (PMC4414138; doi:10.18632/oncotarget.2876)
Supplement: Supplementary file 1 [file oncotarget-06-3578-s001.pdf]

SUPPLEMENTARY FIGURES

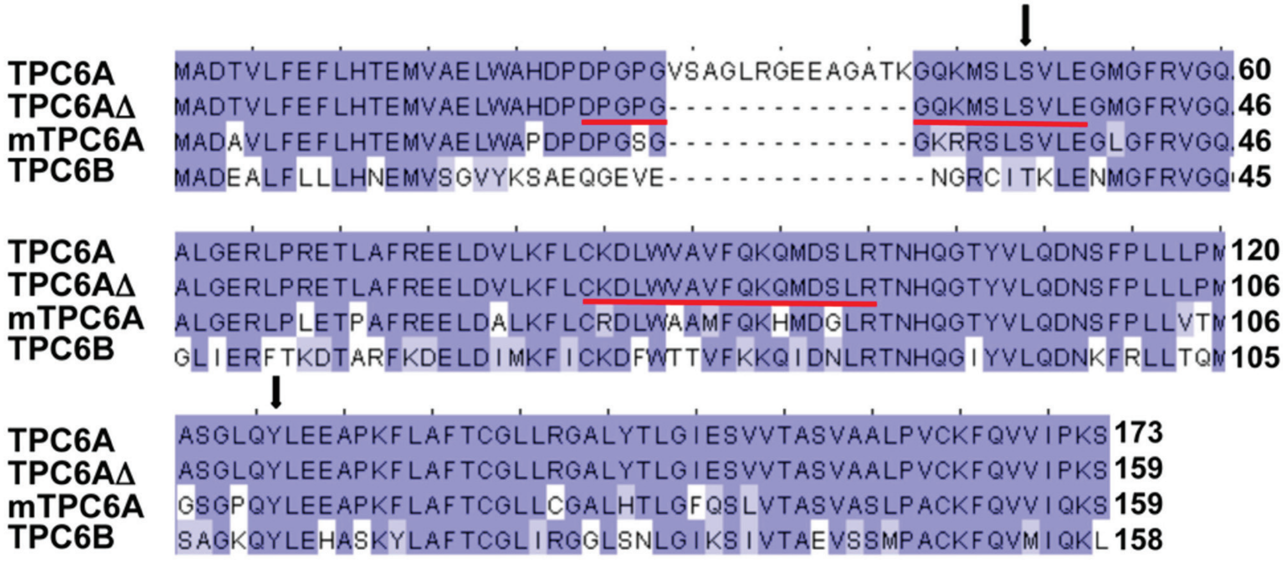

Supplementary Figure 1: Alignment of TPC6A with homologues. TRAPPC6A (TPC6A) or Trs33A2 (173 aa, GenBank accession NP_077013) is a conserved protein from yeast to human. There are two splicing isoforms of TPC6A. The truncated TPC6AΔ or Trs33A1 has a deletion of 14 amino acids at the *N*-terminus at the amino acid #29–42 of the wild type. Murine TPC6A (mTPC6A) (159 aa, NP_080236) shares 95% similarity with TPC6AΔ, and 85% for isoform TRAPPC6B (TPC6B) (isoform 1, 158 aa, NP_001073005). Two potential phosphorylation sites of TPC6A at Ser49 and Tyr126 are predicted (see arrows). These residues correspond to Ser35 and Tyr116, respectively, in TPC6AΔ. Polyclonal antibodies against amino acid segments #24–38 (DPGPGGQKMSLSVLE) and #70–86 (CKDLWVAVFQKQMDSLR), respectively, in TPC6AΔ were made in rabbits (underlined in red). Antibody against phosphor-Ser35 using the amino acid segment #24–38 in the TPC6AΔ as a backbone was also generated, as described (27).

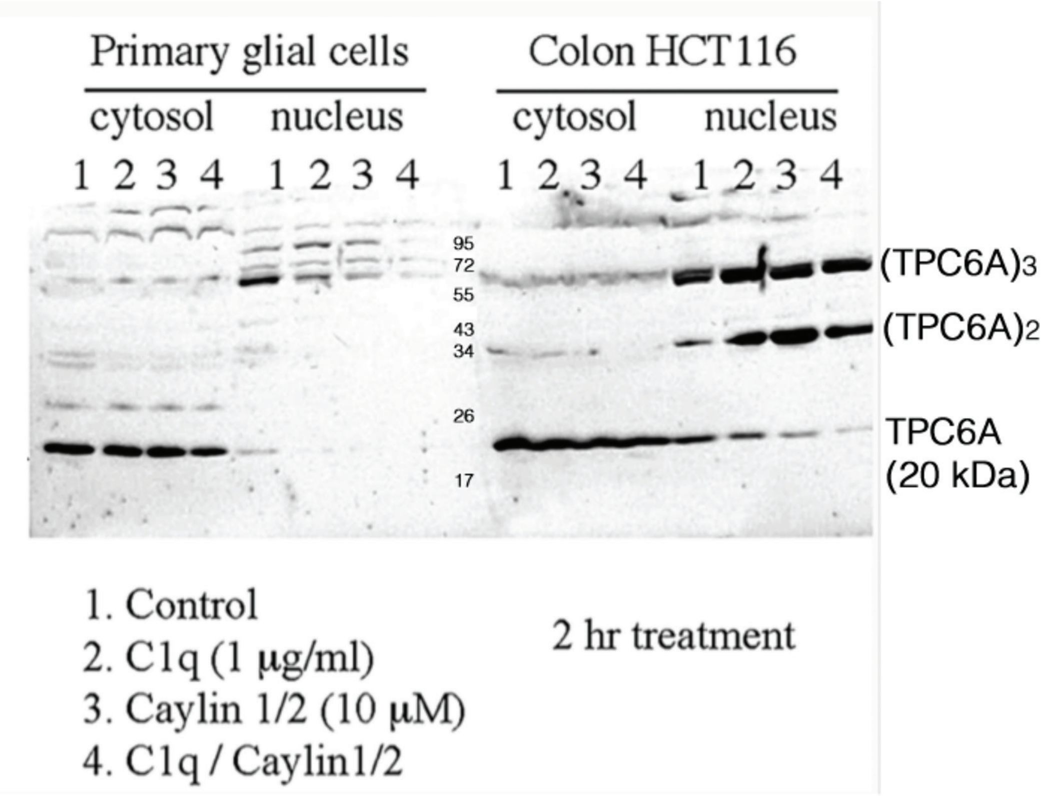

Supplementary Figure 2: High molecular weight TRAPPC6A (TPC6A) complexes in the nucleus. Primary glial cells and colon HCT116 carcinoma cells were treated with purified complement C1q and/or Caylin1/2 for 2 hr, followed by determining the cytosolic and nuclear distributions of TPC6A (20 kDa as monomer) under reducing SDS-PAGE. By Western blotting, presence of dimer, trimer and larger sizes of TPC6A is shown in the cytosol and nuclei. Caylin is an inhibitor of MDM2 or activator of p53. Polymerized TPC6A proteins were not ubiquitinated (data not shown).

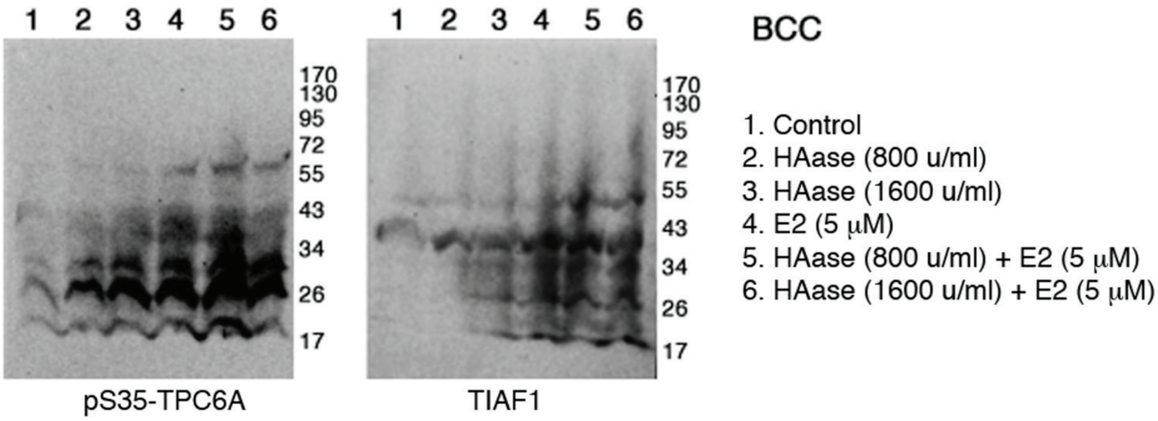

Supplementary Figure 3: Hyaluronidase PH-20 and 17β-estradiol (E2) increase the polymerization of TIAF1 and pS35-TPC6A. BCC cells were treated with PH-20 and E2 for 3 hr, followed by preparing whole cell lysates for Western blotting analysis. Both PH-20 and E2 increased the aggregation of TIAF1 and pS35-TPC6A (reducing SDS-PAGE).

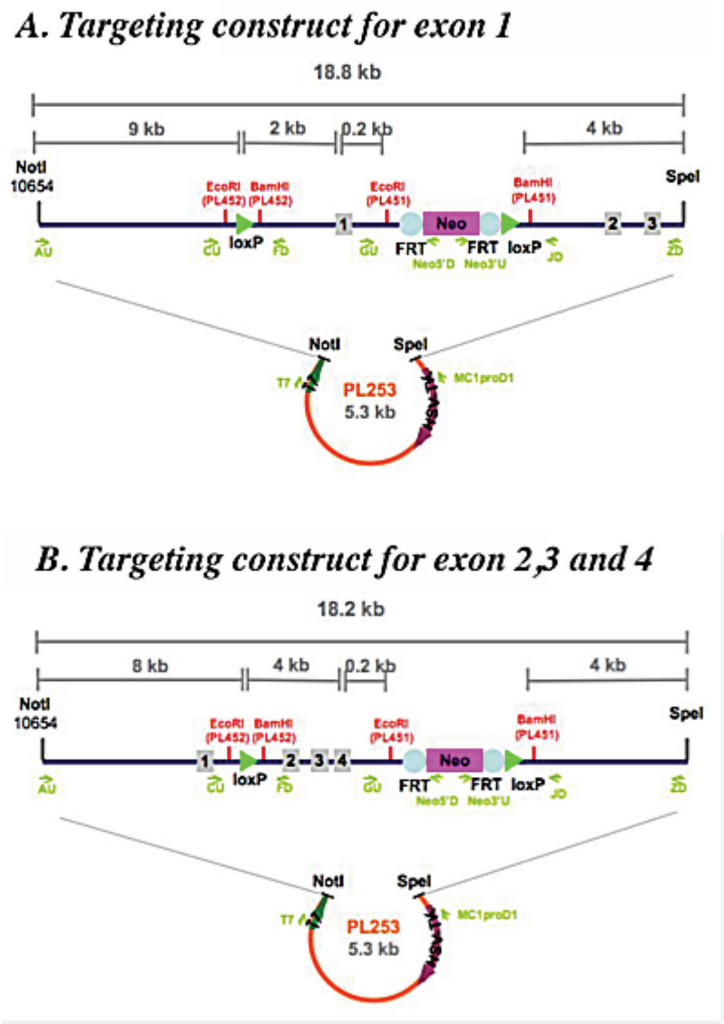

Supplementary Figure 4: Targeting constructs and design.

VIDEO INFORMATION

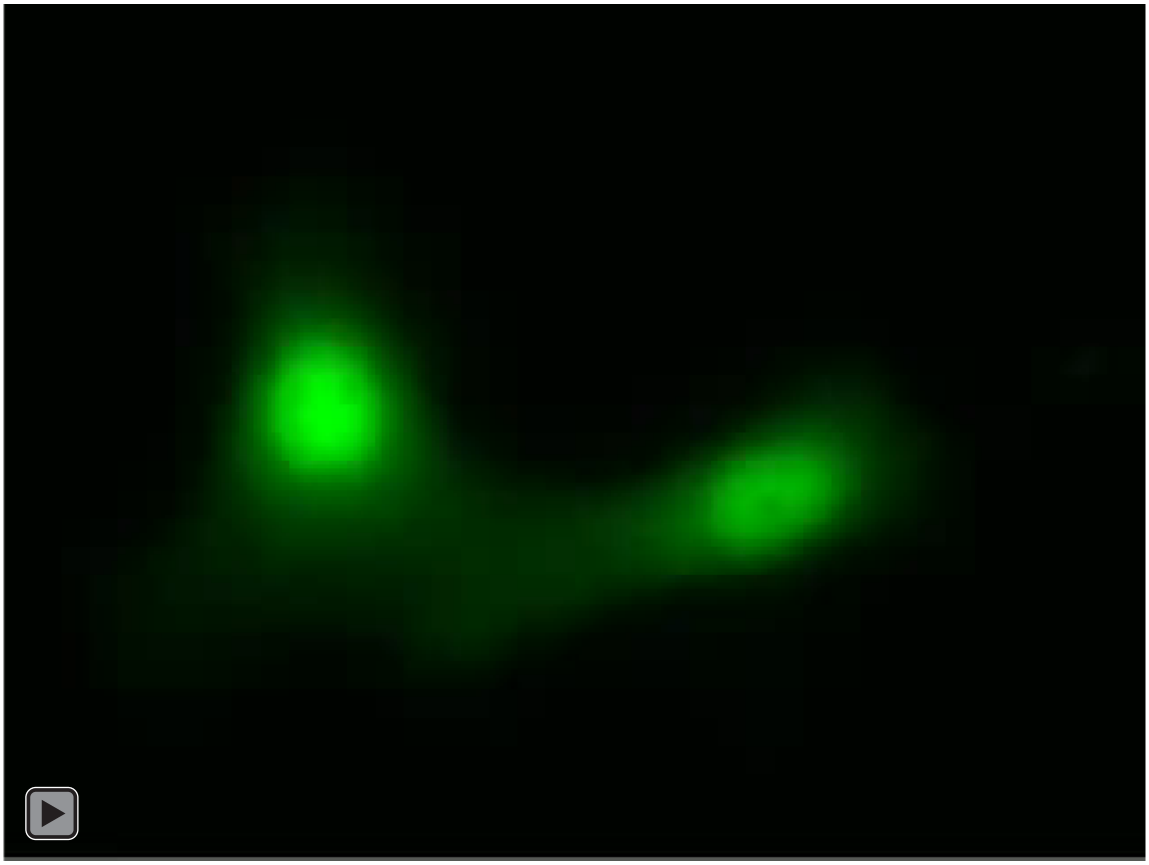

Video 1: WWOX knockdown induces TPC6AΔ aggregation. COS7 cells were transiently overexpressed with EYFP-TPC6AΔ and WWOXsi RNA and cultured for 24 hr. Time-lapse microscopy was then carried out at 37°C in 5% CO2 atmosphere in a culture chamber for 16 hr (30 min per frame). Time-related aggregate formation of EYFP-TPC6AΔ is shown (see cytosolic punctates).

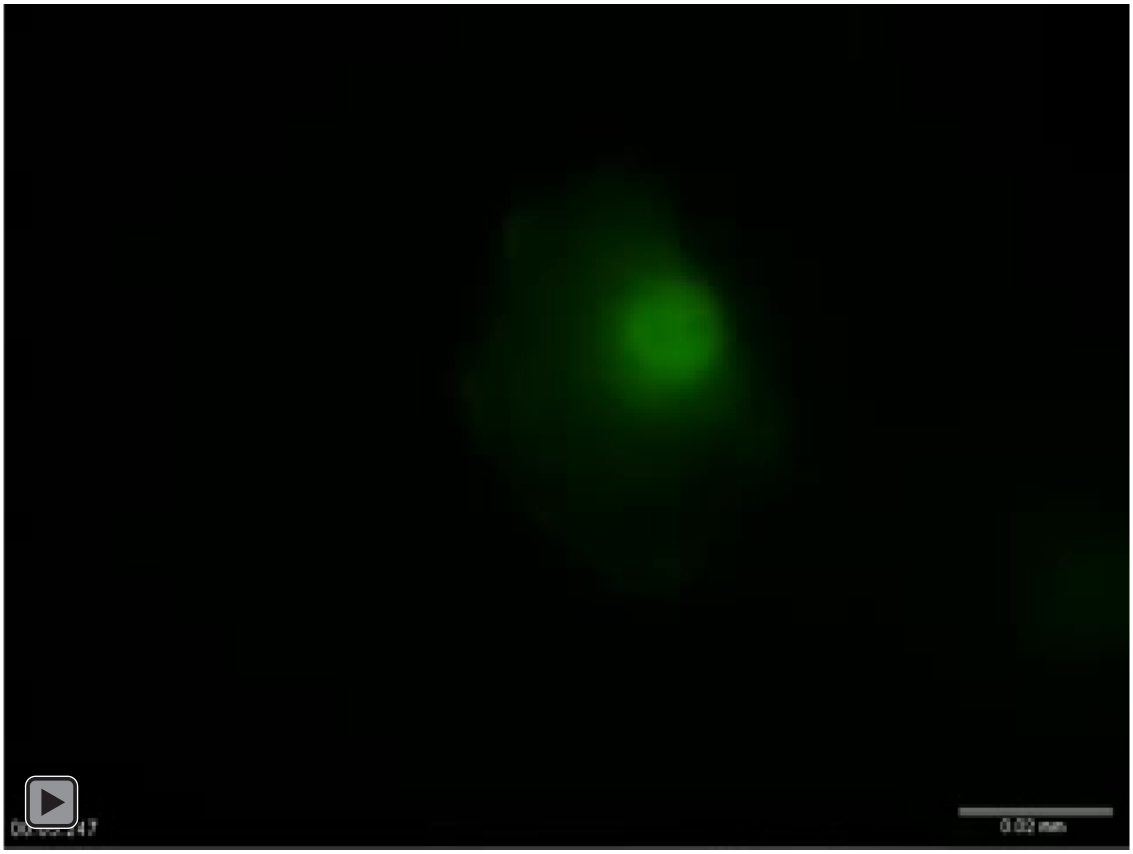

Video 2: Scramble siRNA does not induce TPC6AΔ aggregation. COS7 cells were transiently overexpressed with EYFP-TPC6AΔ and scramble siRNA and cultured for 24 hr. Time-lapse microscopy was then carried out at 37°C in 5% CO2 atmosphere in a culture chamber for 16 hr (30 min per frame). No aggregates of EYFP-TPC6AΔ were generated.
